# D-Shaped Left Ventricle, Anatomic, and Physiologic Implications

**DOI:** 10.1155/2017/4309165

**Published:** 2017-10-08

**Authors:** Eder Hans Cativo Calderon, Tuoyo O. Mene-Afejuku, Rachna Valvani, Diana P. Cativo, Devendra Tripathi, Hans A. Reyes, Savi Mushiyev

**Affiliations:** ^1^Department of Medicine, New York Medical College, Metropolitan Hospital Center, Valhalla, NY, USA; ^2^Cardiology Division, Department of Medicine, New York Medical College, Metropolitan Hospital Center, Valhalla, NY, USA

## Abstract

Right ventricular loading/pressure influences left ventricular function because the two ventricles pump in series and because they are anatomically arranged in parallel, sharing the common ventricular septum. Flattening of the interventricular septum detected during echocardiographic examination is called D-shaped left ventricle. We present a case of an elderly male of African descent, who presented with increased shortness of breath. Transthoracic echocardiogram showed flattening and left sided deviation of interventricular septum causing a decreased size in left ventricle, secondary to volume/pressure overload in the right ventricle. While patient received hemodialysis therapy and intravascular volume was removed, patient blood pressure was noted to increase, paradox. Repeated transthoracic echocardiogram demonstrated less left deviation of interventricular septum compared with previous echocardiogram. We consider that it is important for all physicians to be aware of the anatomic and physiologic implication of D-shaped left ventricle and how right ventricle pressure/volume overload affects its function and anatomy.

## 1. Introduction

Pulmonary hypertension (PH) frequently occurs in patients undergoing maintenance hemodialysis (HD) [1, 2]. Patients with PH may have right ventricular pressure overload manifested as abnormal motion of the interventricular septum, which can be observed on echocardiography [3]. The left ventricle becomes D-shaped, which is a sign of increased preload in the right ventricle (RV) displacing the septum toward the left. Septal flattening is best appreciated in the short-axis view at the level of papillary muscles (basal or mid-left ventricle).

## 2. Case

This is an 87-year-old African male who presented to the Emergency Department complaining of increase shortness of breath after missing 2 sessions of hemodialysis. Past medical history includes end stage renal disease on hemodialysis therapy, heart failure with preserved ejection fraction, permanent atrial fibrillation, severe pulmonary hypertension, and pericardial effusion with placement of pericardial window. Physical examination revealed blood pressure of 117/59 mmHg, heart rate of 91 bpm, respiratory rate of 18 per minute, O2 sat of 96% on room air, and temperature of 37°C. There were internal jugular vein distention, irregular heart sounds, and basilar lung crackles bilaterally. Electrocardiogram (EKG) confirmed atrial fibrillation and right ventricle hypertrophy with right axis deviation. There were normal QRS voltage and complexes, T waves, and ST segments. Laboratory data were remarkable for troponin I 0.04 ng/ml (normal < 0.02 ng/ml), pro-brain natriuretic peptide 108,187 pg/ml, potassium 3.4 mmol/Lt, creatinine 2.8 mg/dl, hemoglobin 11.4 g/dl, total bilirubin 1.41 mg/dl, and albumin 3.1 gr/dl. Cardiomegaly and blunting of the right and left cost phrenic angles were evident on chest X-ray.

**Figure 1:**
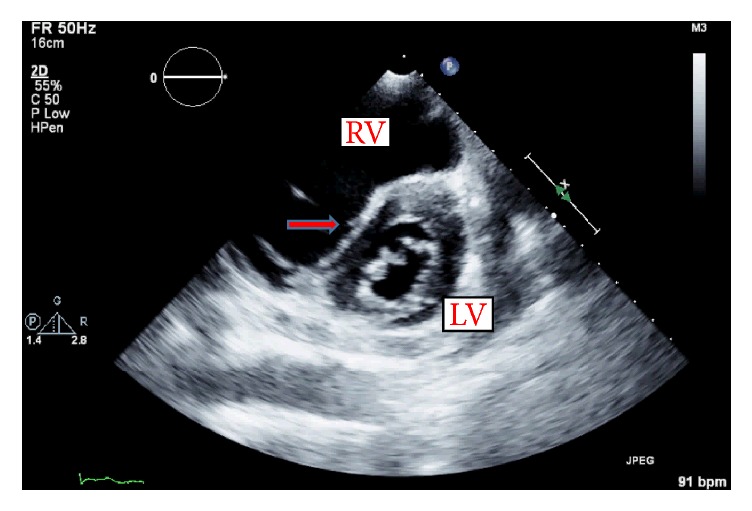
Transthoracic echocardiogram 2D during end-diastolic phase, illustrating flattening of the interventricular septum (D-shaped left ventricle) secondary to right ventricle (RV) overload and increased pressure. Important left deviation of septum into the left ventricle (LV) noted (red arrow).

Patient was admitted to medical intensive care unit (MICU) for fluid overload secondary to missing hemodialysis therapy and severe pulmonary hypertension. While in MICU, transthoracic echocardiogram was done, which revealed normal left ventricular (LV) systolic function (ejection fraction > 55%) and severe tricuspid regurgitation (coronary sinus and right atrium were noted to be dilated, and a small loculated pericardial effusion was seen behind the right atrium, with no evidence of cardiac tamponade. Right ventricular systolic pressure was elevated at >60 mm Hg, consistent with severe pulmonary hypertension, and flattening of the interventricular septum (D-shaped left ventricle) with RV overload (Figure 1).

Shortly thereafter, renal service was consulted and hemodialysis was initiated. Vital signs were recorded and reviewed, while the patient was undergoing hemodialysis therapy, as is illustrated on Table 1, the blood pressure trended up continuously as volume was being removed.

**Figure 2:**
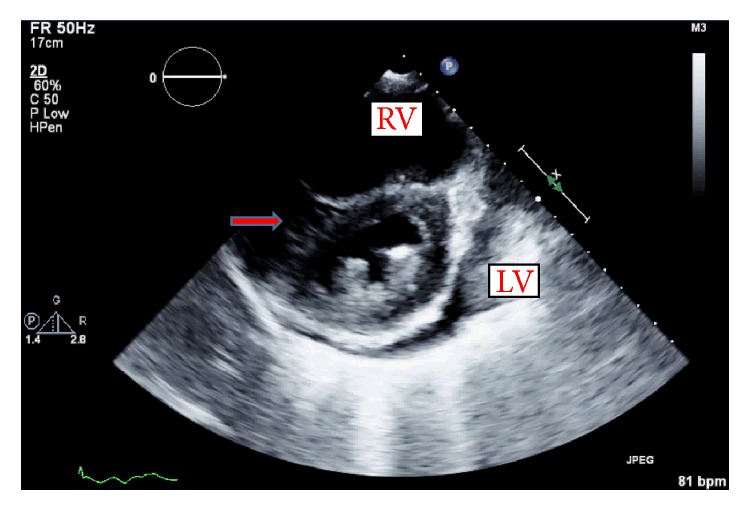
Transthoracic echocardiogram 2D during end-diastolic phase, after hemodialysis showing improvement of flattening (red arrow) of the interventricular septum (D-shaped left ventricle) and displacement toward left ventricle (LV). Right ventricle (RV).

After hemodialysis session, the patient's shortness of breath improved and blood pressure trended up instead down as we may expect in normal individuals after removing intravascular volume. Transthoracic echocardiogram was repeated and demonstrated less left deviation of interventricular septum compared with previous echocardiogram (Figure 2).

Upon discharge, the patient was seen in pulmonary clinic and cardiology clinic as an outpatient at one week and two-month follow-up. He was doing well with improvement of baseline shortness breath and adherence with hemodialysis therapy.

## 3. Discussion

We are describing this case of severe pulmonary hypertension (PH) in an end stage renal disease (ESRD) patient associated with increased right ventricular volume and pressure causing interventricular motion abnormalities demonstrated on echocardiography and paradoxical blood pressure changes after hemodialysis.

PH can be due to a primary elevation of pressure in the pulmonary arterial system alone (pulmonary arterial hypertension; precapillary PH) or secondary to elevations of pressure in the pulmonary venous and pulmonary capillary systems (pulmonary venous hypertension; postcapillary PH). Chronic kidney disease has been associated with development of PH, included in group 5 pulmonary hypertension (multifactorial) as defined by the World Health Organization [4, 5]. It has been suggested that metabolic and hormonal changes in ESRD patients may lead to pulmonary artery stenosis and also pulmonary vascular resistance, ultimately leading to PH. Thus, the etiology of PH in ESRD patients undergoing prolonged hemodialysis may be multifactorial [6].

Echocardiography is an important tool that can easily make an early diagnosis of RV dysfunction and PH, and it also can be very helpful in selecting an appropriate treatment approach to prevent progression of RV dysfunction [7].

**Figure 3:**
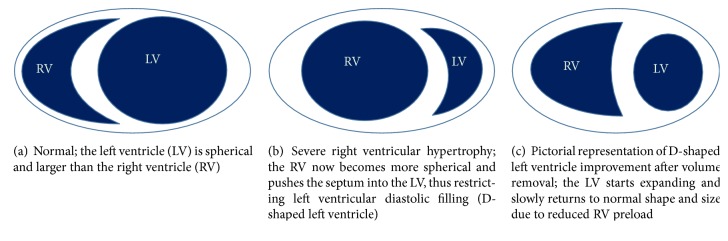
Relation of morphology of right and left ventricle.

Acute and chronic alterations in right ventricular loading influence left ventricular function because the two ventricles pump in series and they are anatomically arranged in parallel, sharing the common ventricular septum (Figure 3(a)). Under these conditions, during the end-diastolic phase there is a leftward displacement of the interventricular septum producing deformity of the short-axis profile of the left ventricle [8] (Figure 3(b)). Systolic overload of the right ventricle produces an inversion of the normal left to right transventricular septal systolic pressure differential. These changes are reversible, as demonstrated by echocardiographic studies of ventricular septal curvature after hemodynamic resolution of acute pressure or volume overload [9] (Figure 3(c)).

In normal circumstances, RV pressure is equal to the pressure in the pulmonary artery. Typically, in RV pressure overload the RV free wall is hypokinetic and is best appreciated from parasternal long axis projections. Normally, the shape of the LV cavity will be circular because of the higher LV pressure throughout the cardiac cycle. However, in the presence of RV pressure overload, such as pulmonary hypertension, the interventricular septum will shift towards the LV and the septum will appear flattened during systole, which is best visualized on the parasternal short-axis view, just below the mitral valve. The higher the RV pressure is, the further the septum will displace into the LV resulting in a D-shaped LV cavity (Figure 3(b)). Of note, septal flattening in the presence of elevated RV pressure should be distinguished from (isolated) RV volume overload, which leads to a septal flattening during diastole [10].

The most common causes of RV volume overload are tricuspid and/or pulmonary regurgitation in the presence of various cardiac pathologies. RV volume overload leads to increase size and expansion of RV; these changes not only produce a displacement of the interventricular septum posteriorly during diastole but also change the shape of the septum and left ventricle. Further, although vigorous contraction of the right ventricle does appear to contribute in some cases to the anterior systolic motion of the septum, the major factor causing paradoxical septal motion appears to be the change in ventricular shape from diastole to systole [11].

Flattening of the interventricular septum detected during echocardiographic examination is called D-shaped left ventricle (Figure 3(b)). D-shaped left ventricle evidenced during systole (particularly end-systole) suggests RV pressure overload, whereas a D-shaped ventricle in diastole suggests RV volume overload. Septal flattening is best appreciated in the short-axis view at the level of the basal or mid-left ventricle. If severe TR coexists with pulmonary hypertension, combined RV pressure and volume overload results in septal flattening occurring in both systole and diastole. Since diastolic interventricular septal flattening represents relative RV and LV filling, an RV overload pattern may be manifest in the setting of mitral stenosis and less severe forms of TR.

Our patient initially was admitted due to worsening shortness of breath secondary to chronic severe pulmonary hypertension and volume overload after missing two hemodialysis sessions. Transthoracic echocardiogram showed severe tricuspid regurgitation, dilated coronary sinus, dilated right atrium, and marked deviation of the interventricular septum into left ventricle.

In healthy individuals, when intravascular volume is decreased, preload decreases, which, in turn, decreases cardiac output and produces a drop in blood pressure. Our patient presented with severe pulmonary hypertension, volume overload, and right ventricle dilation. In some circumstances such as cardiac tamponade and right ventricle myocardial infarction, intravenous fluid (usually isotonic saline) should be given to patients with evidence of low cardiac output (hypotension, hypoperfusion, and a low or normal jugular venous [JVP] pressure) who do not have pulmonary congestion or evidence of right heart failure. Our patient demonstrates RV pressure and volume overload with acute decompensation of right ventricle function (right ventricle failure) in these circumstances volume removal will improve right ventricle function and cardiac output.

As the right ventricle is exposed to increased pressure and volume overloaded, there is a resultant displacement of interventricular septum, leading to decreased size of the left ventricle producing a decline in cardiac output. While the patient was on hemodialysis therapy and fluid was being removed, volume overload in the right ventricle decreased, causing a reduction in RV size and subsequent displacement of the interventricular septum toward the left ventricle. This allowed the left ventricle to expand, fill, and contract better, increasing cardiac output (cardiac output = heart rate × stroke volume) as evidenced by a rising blood pressure. In normal individuals after removing intravascular volume, a decline can be expected in blood pressure, as we illustrated in our patient this effect is paradoxical in those patients with increased right ventricle pressure and volume overload. These findings can be confirmed by echocardiography changes evidenced after hemodialysis, showing decreased displacement of interventricular septum into left ventricle. Although initial right ventricular hypertrophy may be adaptive, in chronic overload states the remodeling process ultimately affects and perhaps irreversibly damages left ventricular function leading to overall decreased cardiac output. We do not recommend to routinely remove fluid in patients who are experiencing RV dysfunction; the decision should be individualized to each patient.

Basic physiology and anatomic relation of left and right ventricle is sometimes forgotten. Thus, it is important for our daily practice to acknowledge how anatomic and physiologic changes on right ventricle may affect left ventricle and normal heart functioning.

More insight into right ventricular hypertrophy and remodeling needs to be determined for developing potential new therapeutics and for improving prognosis.

## Figures and Tables

**Table 1:** Blood pressure monitoring and time relation with hemodialysis.

Before hemodialysis	113/75 mm Hg
During hemodialysis	125/82 mm Hg
After hemodialysis	132/84 mm Hg
